# The hidden world of nanoplastics colliding with neurodegenerative diseases

**DOI:** 10.1172/JCI204824

**Published:** 2026-02-16

**Authors:** Andrew B. West, Matthew J. Campen, Mark Wiesner, Jason A. Somarelli, Jason W. Arnold

**Affiliations:** 1 Department of Pharmacology and Cancer Biology, Duke University Medical Center, Durham, North Carolina, USA.; 2 Department of Pharmaceutical Sciences, College of Pharmacy, University of New Mexico Health Sciences, Albuquerque, New Mexico, USA.; 3 Department of Civil and Environmental Engineering, Duke University, Durham, North Carolina, USA.; 4 Department of Medicine, Duke Cancer Institute, and; 5 Department of Molecular Genetics and Microbiology, Duke University Medical Center, Durham, North Carolina, USA.

Nanoplastics are tiny, irregular plastic fragments that are far smaller and more biologically active than microplastics. They are detected across human tissues, including recent reports of unexpectedly high concentrations in the brain. Early evidence links higher tissue nanoplastic burdens with both cardiovascular and neurological disease, and early experimental models suggest these particles may promote neuroinflammation, impair cognition, and catalyze α-synuclein fibrillization through lipid-like colloidal interactions. Because current detection methods are limited, and exposure sources remain poorly defined, urgent research is needed to clarify how nanoplastics enter the brain, which polymer types pose the greatest risk, and whether they contribute causally to common neurodegenerative disorders, such as Parkinson’s disease and dementia (Figure 1). This Viewpoint reviews this emerging evidence linking nanoplastics to pathways implicated in neurodegenerative disease and discusses key knowledge gaps and priorities for future investigation.

## Exposing nanoplastic presence in biological and environmental settings

Whether an existential threat or a benign nuisance, nanoplastics may be a ubiquitous component of our food, our water, and even our air. Nanoplastics exhibit behaviors distinct from larger microplastics that are visible under standard light microscopy. Owing to their extremely small, fractured, and irregular dimensions, environmental nanoplastics have increased bioavailability, unique surface chemistries, colloidal properties, and increased plastic additive release (1). The extent of nanoplastic formation and proliferation in the environment, and how nanoplastics influence health outcomes, remain unknown. As will be discussed, investigation of these questions remains constrained by pervasive technological limitations, underscoring the need for focused interim appraisals of emerging evidence and research priorities.

Fine particle exposures have long been linked to a variety of adverse health outcomes (2). Unlike many classes of fine particles, nanoplastics are principally composed of carbon chains with similarities to proteins, nucleic acids, and lipids, making them difficult to distinguish from biological materials. In practice, nanoplastics evade all but the most specialized and sensitive means of detection. Nanoplastics are exceptionally durable, reflecting the miraculous engineered resiliency of larger plastic components. While many studies continue to surveil larger microplastics, researchers attempting to measure nanoplastic particles in biological tissues, fluids, and environmental sources liken the experience to finding a *toothpick* in a haystack, distinguished from the comparatively trivial task of finding a *needle* in a haystack. New research shows that some types of nanoplastic particles, similar to those detected in the human brain, might interact with aggregation-prone proteins associated with cognitive impairment and neurodegenerative diseases, catalyzing their conversion toward pathological conformations. Below, we will discuss methodological and technical challenges of investigating the emerging link between nanoplastic exposures and neurodegenerative diseases.

## Challenges of quantifying environmentally derived nanoplastics

Degrading tires, textiles, construction materials, and other consumer products represent major sources of non-exhaust nanoparticles (3). Approximately 70% of commercial plastics are semicrystalline polymers highly susceptible to mechanical failures on the nanoscale level (4). Intrinsic chain scission events lead to the production of nanoplastics (10 nm–1 μm) under quiescent conditions associated with typical tasks in daily life. Although nanoplastic shedding can be difficult to study under controlled conditions, detecting and recovering nanoplastics from biological fluids and tissues is even more challenging. There is no known single technique or measurement possible that simultaneously reports on nanoplastic concentration, size, shape, polymer type, and chemical additive composition. The most informative studies are those that deploy several orthogonal measures that each offer different insights, albeit with their own biases and uncertainty. One chemical principle widely employed to find the proverbial toothpick in the haystack is the presumed resiliency of nanoplastics to chemical degraders, such as strong acids, bases, enzymes, and oxidizers, that otherwise digest away biological material.

In 2022, the first evidence of plastic pollution in human blood was provided primarily through a pyrolysis mass spectrometry method, relying on fingerprint ion trace intensities relative to pristine plastic standards. Alarming levels of plastic pollution were identified in many young adults, though some blood samples had no quantifiable plastic (5). Subsequent studies from independent groups highlight the ongoing technical challenges in arriving at a consensus for the detection and measurement of certain polymers in blood (6, 7). Whether nanoplastics are introduced into circulation through ingestion, inhalation, or combinations of both, several subsequent studies have quantified nanogram to microgram levels of plastic per gram of tissue or biofluid evaluated. In each study, wide variabilities in plastic levels between individuals challenge associations that might otherwise explain variances. As such, no known or measured covariable explains sample heterogeneity in plastic concentrations to a satisfying degree. Individual (or tissue) age appears not to significantly predict plastic particle concentrations in tissues or fluids.

Because so little is known regarding exposure, distribution, and elimination of nanoplastics from the human body, evidence-based recommendations on how the public might avoid environmentally derived nanoplastics cannot be made at this time, and claims made to the contrary should be met with appropriate skepticism.

## Nanoplastic reservoirs in the human brain

In 2025, initial measures of nanoplastics in human brain tissues were disclosed, together with measures in kidney and liver (8). Electron microscopy and spectroscopy measurements corroborated mass spectrometry estimations of polymer types. After alkaline digestion of biological materials and dispersal of resilient materials into solvents, nearly all plastic particles in the brain presented crystalline shards, largely with dimensions less than 200 nm in length and 40 nm in width. Although the extraction process itself may generate agglomerated fragments, the resulting particles are morphological and spectrally similar to nanoplastics associated with quiescent release from commonly used plastics worldwide (4). As the field rapidly evolves, methodologies and quantification techniques between studies differ to the point where direct comparisons of concentrations of plastics from one study to the next may lead to spurious conclusions. Studies reliant at least in part on pyrolysis mass spectrometry to extrapolate absolute quantities (e.g., micrograms per gram) of plastic contaminants in biological samples have been criticized for possible overestimations of certain polymer types (9). On the other hand, the proportion of fractured environmental nanoplastics that survive through the degradation process necessary for biological samples is not clear, leading to possible underestimations of certain labile polymer types. As will be discussed further, absolute quantities of plastic contaminants may not be as important as other properties known to strongly influence toxicity including particle size, surface charge, and compositions of additive release.

Presently there are three important conclusions that appear to be broadly supported: (a) Human age does not predict plastic pollution concentrations in tissues and fluids. While it might be presumed that individuals acquire more plastic in tissues with age, this is not supported by evidence; on the contrary, there is no correlation between human age and plastic contaminants accumulating in tissues or fluids. (b) Increased concentrations of plastic have been measured in worse disease states. For example, patients undergoing carotid endarterectomy for asymptomatic carotid artery disease with measured plastics in artery plaques had a higher risk of major adverse cardiovascular events than people with plastic contaminants below quantifiable levels. Brain tissues affected by dementia (e.g., Alzheimer’s disease or vascular dementia) had higher levels of plastic contaminants than neurologically normal controls (8, 10). Correlation is not equivalent to causation, and models must be employed to clarify the role of nanoplastics in disease mechanisms. Because nanoplastics are not yet routinely measured in environmental samples, there are no geographic or exposure trends to support epidemiological studies currently. (c) Concentrations of plastic appear to be increasing in tissues and fluids from biospecimens taken from recent years compared with older samples collected in the past under the same conditions and protocols. The rate of increase in plastic contaminants in biological samples appears to roughly correspond with the rate of increase of plastic contaminants in the environment, mirroring the increase in global plastic waste production.

The properties of environmentally relevant nanoplastics that might lead to brain tissue accumulation and penetration through protective barriers, like the blood-brain barrier in humans, are unknown. There is acute cause for concern for human brain health with respect to exponentially rising levels of nanoplastic pollutants in our environment. Studies in seabirds found that birds with higher levels of ingested plastic waste demonstrated proteomic changes in the brain associated with neurodegenerative diseases (11). In the laboratory, oral feeding of environmentally realistic polyethylene nanoplastics to mice led to inflammation and striking cognitive impairment (12). Most model studies utilize commercially available polystyrene nanobeads as exemplary nanoplastic contaminants. However, polystyrene may not be accumulating in human brain tissue, and engineered spherical nanobeads used in research bear little similarity to crystalline-plastic shards recovered from brain tissues. Better knowledge of the properties of nanoplastics that lead to brain penetration in both health and disease, including the effects of polymer type, charge, shape, and size, and what types of cells in the brain harbor nanoplastics, will guide effective experimental designs.

## Identifying sources of nanoplastics in the brain

With so little yet known in this emergent field, there is an urgent need to implement monitoring programs to track nanoplastics in environmental samples and understand the biological impacts of these materials. A key lesson learned from research on engineered nanoparticles is that physiological and environmental transformations of these materials may greatly alter their bioavailability and toxicity (13, 14). According to national and international databases of publicly funded research efforts, there are egregiously few ongoing efforts attempting to measure nanoplastics in environmental or dietary samples and fewer studies still to understand overall biological effects of environmentally relevant nanoplastics. A promising and potentially transformative technique applying hyperspectral stimulated Raman scattering measurements in fluids identifies plastic particles as short as 100 nm, i.e., including the size range of nanoplastic particles known to accumulate in the brain (15). Using this approach, greater than 10^5^ plastic particles per liter, on average, were found in bottled water from three different brands acquired from large retailers (15). Another study employed a suite of biophysical measures including nanoparticle tracking, dynamic light scattering, electron microscopy, and attenuated total-reflection Fourier transform infrared (ATR-FTIR) for polymer identification, identifying over 10^9^ nanoplastic particles per milliliter, composed primarily of polypropylene, produced by steeping teabags purchased from popular online outlets (16). In both these studies, the properties associated with the discovered nanoplastics are compatible with properties of nanoplastics identified in the brain.

While there is some traction in microplastic and microfiber pollution tracking, it should be emphasized that the relative contribution of larger bio-unavailable microplastics to global nanoplastic accumulations is not known. Assuming nanoparticle inhalation as a major pathway underlying nanoplastic accumulation in the brain, a 2024 systematic review indicates that shared indoor air environments can lead to shared nanoplastic exposure among individuals in the same space (17), though establishing bioaccumulation of nanoplastics in shared environments will be important to study. It is hoped that emerging technologies will be capable of informing on nanoplastics on a broader scale in the near future. Uncertainty about actionable strategies to prevent nanoplastic accumulation in the brain creates opportunities for misinformation and exploitation. Fraudulent commercial services are emerging, claiming technologies that detoxify nanoplastics from the body, as well as surveil various household sources of nanoplastics.

## Nanoplastics are colloids linked to neurodegeneration pathways

Nanoparticles tend to disperse through fluids and do not settle under gravity. Nanoplastic mobility, stability, surface functionalization, and environmentally acquired coronas are governed by these size-dependent colloidal behaviors. In the case of neurodegeneration-linked pathways, through a combination of electrostatic interaction, van der Waals forces, and hydrophobic attraction, a strong interaction has been found between polyethylene and polystyrene surfaces and the protein α-synuclein, such that the plastics catalyze protein conformational changes to those associated with propagative pathology in Parkinson’s disease and dementia (18–20). α-Synuclein misfolding in disease is hypothesized to result from aberrant interactions with damaged lipids. Some types of nanoplastic surfaces, which are strongly resistive to biological degradation, may structurally mimic those of disease-associated lipids. Alternatively, perhaps in disease states, nanoplastics directly acquire lipid coronas in disease to facilitate biological transport and pathway interactions. These results support the idea that absolute concentrations of nanoplastics in biological systems may be less important than polymer properties such as size, charge, and corona composition.

Lipid changes are associated with inflammation and neurodegenerative diseases, and lipid droplets of similar sizes to nanoplastics found in the brain are known to accumulate in microglia and correlate with dysfunctional and pro-inflammatory states in dementia (21). Whether nanoplastic accumulations or compositions differ between brain tissues filled with α-synuclein aggregates, tau tangles, or amyloid-β plaques compared with neurologically healthy controls is not yet known but will be critical to discover. Models of disease will help inform whether environmentally realistic concentrations of physiologically relevant nanoplastics harbored in the brain play a causative role in neurodegenerative diseases, have a modifying role in progression, or simply act as benign bystanders. Although it will be difficult to separate effects on inflammation from the propagation of misfolded proteins, it will be critical to employ nanoplastic particles that more closely resemble those found in the human brain and reflect relevant exposures.

## Funding support

This work is the result of NIH funding, in whole or in part, and is subject to the NIH Public Access Policy. Through acceptance of this federal funding, the NIH has been given a right to make the work publicly available in PubMed Central.

NIH grant R01NS064934 to ABW.
NIH grant R01ES014639 to MJC.
National Science Foundation grant 2428209 to JAS.

## Figures and Tables

**Figure 1:**
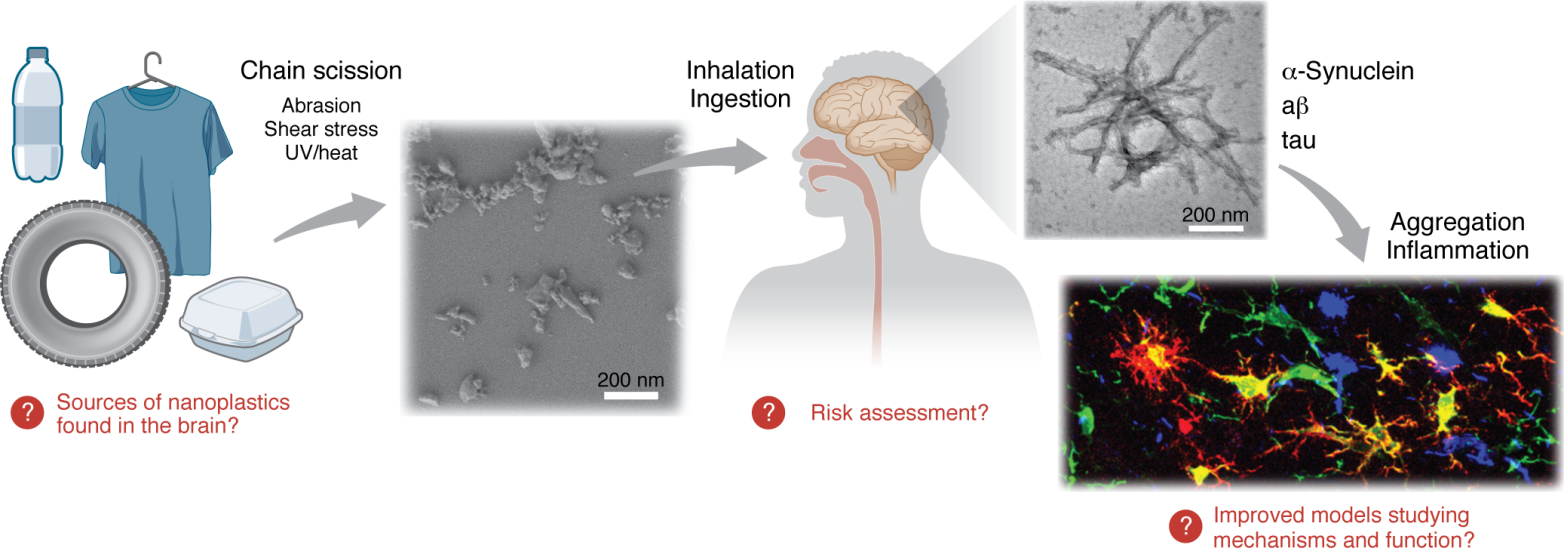
Nanoplastics in the environment and brain and their links to neurodegenerative disease. Degradation of plastic-containing products, including food and beverage containers, clothing, construction materials, and rubber tires, occurs when the semicrystalline polymers comprising the majority of commercial plastics accumulate chain scission events resulting from abrasion, shear stress, and UV/heat exposure. These deteriorating polymers produce micro- and nanoplastics, the latter of which is represented in the electron microscopy image. Inhalation of airborne nanoplastic particles and ingestion of nanoplastics in food and beverages are two routes of entry into the human body, where nanoplastics have been identified in nearly every tissue, including in the brain. The presence of nanoplastics in brain tissue is linked to pro-inflammatory states and aggregation of proteins such as α-synuclein, amyloid-β, and tau that propagate in neurodegenerative diseases. Notably, the size and lipid-like biophysical properties of nanoplastics are conducive to pathological interactions with these proteins. Red question marks highlight priorities for future investigations of nanoplastic reservoirs in the brain.

## Version 1 02/16/2026

Electronic publication
